# Patterns and Factors Associated with Self-Medication among the Pediatric Population in Romania

**DOI:** 10.3390/medicina56060312

**Published:** 2020-06-25

**Authors:** Petruța Tarciuc, Ana Maria Alexandra Stanescu, Camelia Cristina Diaconu, Luminita Paduraru, Alina Duduciuc, Smaranda Diaconescu

**Affiliations:** 1 Family Medicine, University of Medicine, Pharmacy, Science and Technology “Emil Palade”, 540142 Târgu Mures, Romania; petrutatarciuc@yahoo.com; 2 Department of Internal Medicine, University of Medicine and Pharmacy “Carol Davila”, 020021 Bucharest, Romania; alexandrazotta@yahoo.com (A.M.A.S.); drcameliadiaconu@gmail.com (C.C.D.); 3 Internal Medicine Clinic, Clinical Emergency Hospital of Bucharest, 014461 Bucharest, Romania; 4 Mother and Child Department, “Grigore T. Popa” University of Medicine and Pharmacy, 700115 Iasi, Romania; turti23@yahoo.com; 5 Faculty of Communication and Public Relations, National University of Political Science and Public Administration, 012104 Bucharest, Romania; alina.duduciuc@comunicare.ro

**Keywords:** children, self-medication, risks, beliefs

## Abstract

*Background and objectives:* Self-medication is a global phenomenon in both developed and emerging countries. At present, data regarding the practice, patterns, and factors associated with self-medication in Romanian patient groups of various ages and health are relatively scarce. A pilot study that uses a questionnaire was conducted to observe the attitudes as well as the behaviors of a group of Romanian parents related to self-medication, specifically their beliefs and perceived risks of the administration of medicine to their children without medical advice, frequency of self-medications, symptoms, and types of medications most commonly used without medical advice. *Materials and Methods:* The questionnaire was sent via e-mail or WhatsApp link on a mobile phone using the existing data at the general practitioner’s office together with the protection of data form and the informed consent form; some participants completed the questionnaire when they came for a regular visit at the general practitioner’s office. Of 246 applied questionnaires, we had a rate of responses of 98%. *Results:* We found a high percentage (70%) of parents who self-medicate their children. The data reveals a significant relation between parents’ beliefs on self-medication and their tendency to administrate drugs to their children without medical advice. A significant relation was also found between the likelihood of parental self-medication for their children and the number of illnesses experienced by their children over the six-month period prior to the survey. Even when parents have a correct understanding of self-medication risks, these are not aligned with actual behavior; therefore, parents continue to administer drugs to their children without medical advice. *Conclusions:* Our study helps to describe the patterns of parents’ decisions about self-medicating their children and to identify parents who are more predisposed to administering self-medication to their children.

## 1. Introduction

Self-medication is defined as “the taking of drugs, herbs or home remedies on one’s own initiative, or on the advice of another person, without consulting a doctor” [1] or by “the use of medicinal products by the individual to treat self-recognized disorders or symptoms” [2]. In the 1990s, the first worldwide reports of this phenomenon were made, and in 2000, the WHO published “guidelines for the regulatory assessment of medicinal products for use in self-medication” [3]. In pediatrics, self-medication means that medications for various disorders are administered by the caretakers of children without medical consultation. In addition, various reports highlight the fact that teenagers self-medicate on their own [4,5]. Economic, political, and cultural factors are responsible for the prevalence of, and increase in self-medication practices worldwide. These factors include the wide availability of drugs, improper advertising, parental education level, socioeconomic status, and access to healthcare services [2]. Studies reveal an increased prevalence of self-medication among pediatric populations worldwide: Germany 25.2%, China 62%, Italy 69.2%, and France 96% [6,7]. Studies in Pakistan showed that almost half of parents self-medicated children between ages 1 and 5 years, and one-third self-administered medication to children aged 5–12 years [8]. In Romania, it is estimated that 10% of the systemic antibiotics sold at pharmacies are sold without a prescription, and in our country, sales of self-prescribed medications were 488.8 million Euro in 2017; this amount included 103.1 million Euro sales in analgesics and 145.3 million Euro sales in cough and cold products [9,10]. Other studies report that Romanian pharmacists are influenced by the socioeconomic condition of the patient and are more likely to sell antibiotics without a medical prescription to socially vulnerable persons [11,12].

Despite these facts, in a study from Mureş County, Romania, the majority of respondents had good knowledge of antibiotic usage and the risks of self-prescribed antibiotics [13]. A study from another region of Romania regarding self-medication with analgesics showed that 84.8% of 461 adults aged 20–90 years used this type of medicine, both in rural and urban areas, with a predominance of females (75.5%) and young adults (70.1%) [14]. Data regarding self-medication in children by their parents or self-medication of teenagers are scarce in Romania. One study from the western part of the country reported a high prevalence of self-medication in children under 12 years old (81.0%); young mothers (30–39 years old) who were highly educated were most likely to administer medication to their children using the family pharmacy kit (83.6%) for fever, pain, and cough [15]. In our country, the few existing studies report regional data, address different age groups (mainly adults), and focus on specific categories of medication.

This study aimed to identify the individuals’ attitudes and behaviors in regards to self-medication, particularly parents’ beliefs and perceived risks in terms of administering certain treatments to their children without seeking medical advice. We tried to identify behavior patterns connected to medication and self-medication, the types of symptoms that most frequently lead to self-medication, the categories of administered substances, and the relevant context thereof (for instance, outside the hometown).

## 2. Materials and Methods

Data collection: The survey was conducted online, between August and October 2019, using the databases of four family medicine offices in the metropolitan area of Bucharest. The respondents (parents) were also recruited when they came to the doctor’s office for their own medical problems.

Inclusion criteria: Adults enrolled in one of the four family medicine offices with whom the coordinator of the study collaborated with; adults with at least one child aged 0–18 years registered at the same office.

Exclusion criteria: Adults without children; adults with all children older than 18 years.

The study sample comprised a total of 241 adults from various regions of Romania; their children were born in different maternity hospitals, and at the time of the study, they all lived in Bucharest together with their parents.

Instrument questionnaire. The survey questionnaire consisted of 25 questions (Supplementary Text S1) that gathered the opinions of parents on topics related to pediatric self-medication as follows: Frequency of medication without medical advice (measured on a three-point scale, where 3 = often, 2 = sometimes, 1 = never), types of symptoms in which self-medication was given (multiple-choice question), types of medicines parents used for their children (multiple-choice question), the type of online or offline source of medical information (multiple-choice question), and the perceived risks of self-medication (closed question). The beliefs of parents on pediatric self-medication were measured through a question where patients expressed their agreement on a three-point scale (where 3 = disagree, 2 = agree, 1 = don’t know/can’t answer) with a set of statements reproducing beliefs on self-medication derived from literature research.

Categorical data, such as socio-demographics (age, gender, education level, employment, number of children), were also collected during the study.

The questionnaire was sent via e-mail or WhatsApp link on a mobile phone using the existing data at the general practitioner’s office together with the protection of data form and the informed consent form; some participants completed the questionnaire when they came for a regular visit at the general practitioner’s office.

The respondents received the link on WhatsApp or e-mail so as to respond to the survey instrument (the questionnaire) according to their willingness. The respondents were informed that our study did not collect personal data of patients (such as name, address, email, or current state of health). The questionnaire was administered online, i.e., patients received the link with the questionnaire and were solicited to answer the questions therein. As participation in the study was voluntary, the respondents could exit the questionnaire if they did not wish to complete it. The study was approved by the ethics committee of the Romanian Academy of Medical Sciences (no. 2 SNI/27.02.2019).

Statistical analysis was conducted using SPSS 20 software, including descriptive (frequency and percentage) and cross-data (Fisher’s Exact Test) statistics, when applicable. As our study was a preliminarily one and the sample was homogeneous in terms of categorical variables (Table 1), we chose to analyze the overall data, looking at the occurrence of self-medication in general. Fisher’s exact test was performed to determine associations between the frequency of self-medication and the parents’ beliefs regarding medication of their children without medical advice. Furthermore, we used Fisher’s exact test to observe if the likelihood of parental self-medication is related to the number of illnesses experienced by their children over the six-month period prior to the survey.

## 3. Results

The subjects of the current study were 241 adults from an urban area (Bucharest, Romania) whose children were registered in the databases of four family physician offices in Bucharest. Of the 246 applied questionnaires, 241 parents gave their consent to answer the questionnaire. Only 5 parents from 246 declined the questionnaire (response rate 98%).

The socio-demographic characteristics of the sample are presented in Table 1.

### Prevalence and Characteristics of Self-Medication Practices

The majority of parents were members of an online group for medical discussions (62%). The majority of respondents (70%) resorted to self-medication often (9%) or sometimes (61%).

**Figure 1:**
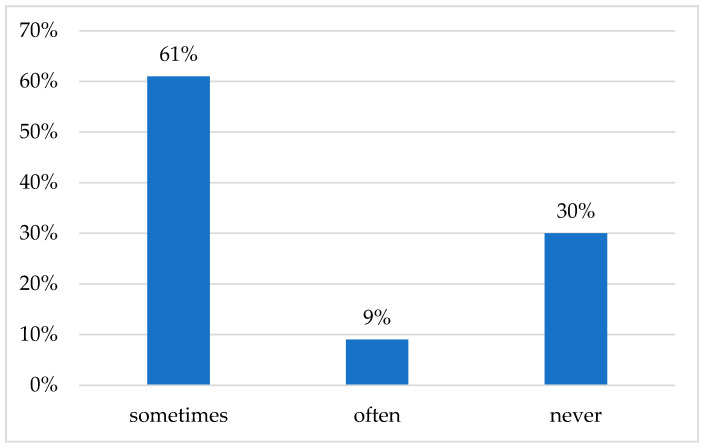
The frequency of self-medication administered to children (*N* = 241 parents).

Of the total sample, 73 persons (30%) reported never having given their children any medicine without prior medical advice (Figure 1).

Parents most often chose to treat their children’s medical issue on their own during the night (53%), when they were unable to access healthcare services (61%), when they were out of their hometown (47%), and during weekends (28%).

**Figure 2:**
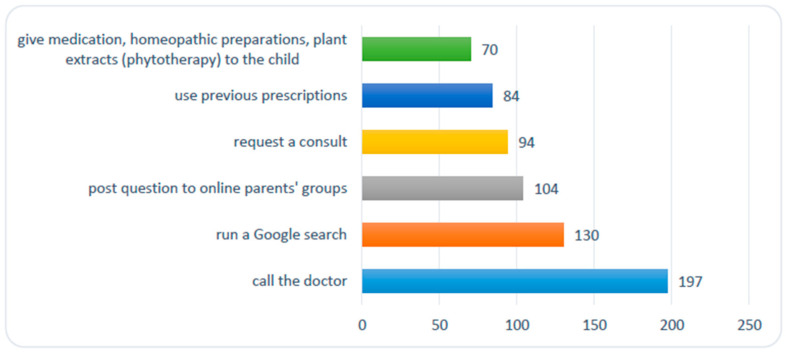
Number of parents that resort to certain solutions when their child is ill.

When their children are ill, a large proportion of our respondents said they call their doctor (N = 211; 88%); only 24 (10%) of the respondents rarely phone the doctor while 6 (2%) of them have never done so (see question 10 from Supplementary Text S1). Some parents (40%) request and receive a medical visit. Among them, there are parents that resort to self-medication using medicines, homeopathic preparations, or plant extracts (phytotherapy) based on personal experience (30%), or that use previous doctor-recommended treatment schemes (35%). Their children’s illness also leads some parents to seek medical information online using Google’s search engine (54%), online parent groups, or medical websites for parents (43%) (Figure 2).

**Figure 3:**
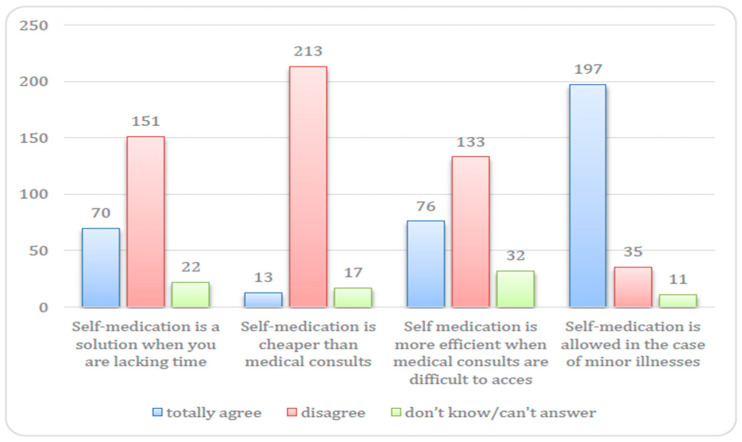
Number of parents that agree or disagree with statements on self-medication.

The results revealed that, in general, respondents are aware that self-medication is undesirable and most (82%) believe that administering certain treatments to their children without a medical consult is only allowed in the case of minor symptoms. Most respondents disagree with the following statements: “Self-medication is cheaper than medical consults” (88%) and “self-medication is a solution when you lack time” (62%). However, around one in three respondents (32%) found self-medication more efficient when medical consults are difficult to access (32%), and 13 respondents (5%) found self-medication to be less expensive than medical consultation (Figure 3).

In our study, we were interested to see if there is a statistically significant association between beliefs regarding self-medication and associated behaviors, as well as whether the incidence of self-medication is higher among parents dealing more often with their children’s health issues. For this purpose, we performed Fisher’s exact test to determine whether the frequency of parents toward self-medication correlated with the frequency of their children’s health issues over the last six months. The results revealed a significant relation between the likelihood of parental self-medication for their children and the number of the children’s health issues (*p* = 0.004) (Table 2), that is, parents that were confronted with more than two medical issues of their children tend to give them drugs without medical advice compared with those who experienced less than two health issues.

We were also interested to see if there is a significant association between the frequency of self-medication and parents’ beliefs regarding medication of their children without medical advice. The Fisher’s exact test values indicated that there is a statistically significant association (*p* = 0.000); specifically, parents who agreed that self-medication is allowed in the case of minor illnesses were the ones that resorted more often to self-medication when dealing with their children’s diseases (Table 3).

We also found that parents who often resort to self-medication of their children were those that believed that self-medication is a solution when health services are difficult to access (*p* = 0.000) and when they do not have enough time (*p* = 0.020) compared with those that do not self-medicate their children (Table 4 and Table 5).

Further, in our study, we tested the relation between self-medication beliefs and ways of purchasing medicine. Considering the descriptive data (Table 6), parents who agreed that self-medication is allowed in the case of minor health conditions are inclined to purchase medicine directly from the pharmacy (*N* = 189; 81%) while parents that disagreed procure the medicine using previous prescriptions (*N* = 33; 20%). We also performed a Fisher’s exact test, which indicates there is no significant difference between those that agree or disagree with the statements regarding self-medication and their means to procure medicine.

The majority of respondents (*N* = 171; 74%) said that they administer drugs when they are familiar with symptoms, a quarter of them (*N* = 61; 25%) when the first symptoms occur, and 34 of the parents (14%) when they notice a worsening of the child’s general condition.

Respondents were aware of the risks of administering medication without a medical prescription. The majority believed that administration errors may occur in terms of both the preparation (77%) and the dosage (60%). Varying numbers of respondents were aware of the possibility of side effects from the medications (51%), the fact that self-medication can mask the symptoms of a severe condition (64%), the likelihood of drug interactions with the onset of adverse effects (38%), and that delay in seeking medical consultation entails risks (38%).

**Figure 4:**
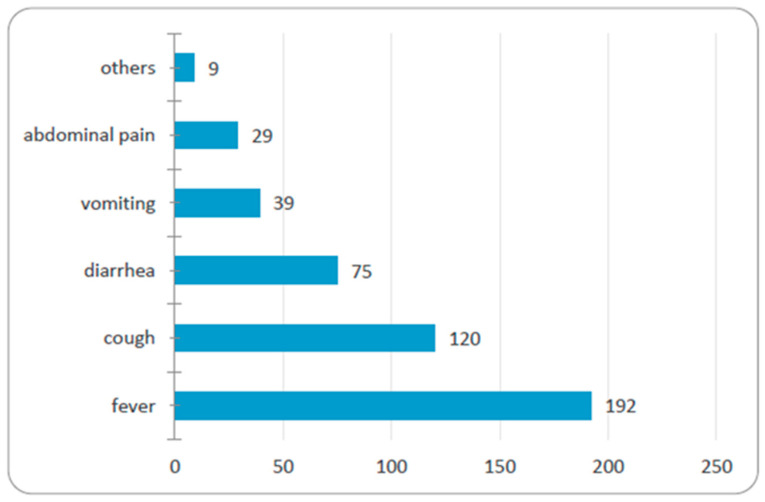
Number of parents that resort to self-medication when they observe the symptomatology of children.

Symptoms that lead parents to treat their children without a medical consult include fever (80%), cough (50%), minor trauma (41%), diarrhea (31%), vomiting (16%), and abdominal pain (12%) (Figure 4).

**Figure 5:**
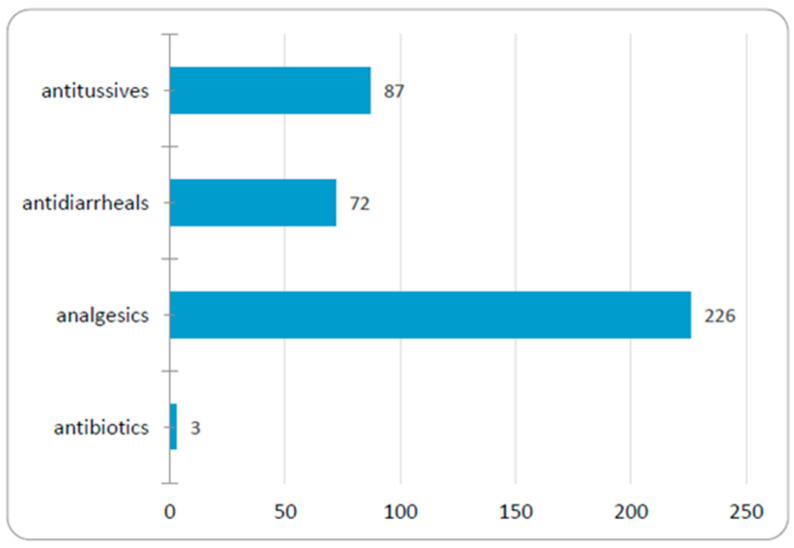
Number of parents that give certain medicine to their children without medical advice.

The medications with which respondents most frequently self-medicate their children are analgesics (94%), antitussives (36%), and antidiarrheals (30%); only 1% of respondents admitted to self-administering antibiotics to their children (Figure 5). The majority of parents (*N* = 233; 95%) stated they purchase medication “directly from the pharmacy,” while others resort to previous prescriptions (*N* = 5; 2%) or to their friends (*N* = 3; 1%).

Our research also explored whether there is a relevant association of self-medication to the use of media versus interpersonal sources of communication for medical purposes. Our results revealed that “traditional” medical sources continue to be frequently accessed by parents to retrieve medical information, namely doctors (98%) and pharmacists (37%). Only 5% reported relying on friends for medical information. Online resources have become increasingly accessible with the large-scale use of new Internet-connected devices. Respondents reported using medical websites (19%), Google (9%), and online parent groups and forums (4%).

**Figure 6:**
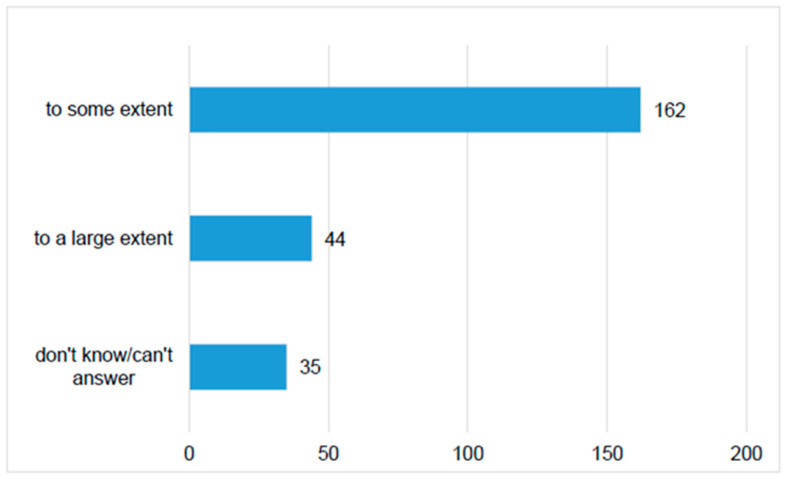
Number of parents that perceived the efficiency of online medical information.

The majority of respondents (67%) believed that online healthcare-related information assisted them to some extent in solving their children’s health issue, while approximately one-fifth (19%) of the respondents considered they found a solution for their medical issue using online sources (Figure 6).

When searching online sources, the majority of respondents (78%) sought information on the most frequent symptoms of common pediatric diseases. Approximately half of the subjects searched for information on therapeutic options for various common pathologies (47%). More than half of the respondents (60%) expressed their interest in homeopathic preparations or plant extracts (phytotherapy), while half of the participants (50%) were interested in other parents’ online descriptions of their experiences with their children’s health issues.

## 4. Discussions

In our study, we found that married urban females with higher university or post-graduate education who join online groups for medical discussions were more likely to self-medicate their children. The high percentage of current Romanian respondents (70%—as 61% who sometimes self-medicated their children and 9% that did the same often) we found who medicate their children without professional medical input is consistent with previous studies conducted in Italy and Pakistan but higher than those in France, Germany, and Spain [6,7,8,16,17]. This variability can be explained by the data collection methods, cultural factors, availability of pediatric services, the costs of healthcare services, and respondents’ socioeconomic status [17,18,19].

The large majority of parents in our study (88%) declared that they call their child’s doctor when the child has an illness (as the responses to question 10 indicate, see Supplementary Text S1). Moreover, when we measured their beliefs regarding self-medication, we found that parents, in a close percentage (82%), believed that self-medication is used only for minor complaints of the children (as the responses to question 20 indicate, see Supplementary Text S1). The certain responses we obtained in our study—the high likelihood of self-medication of children by their parents (70%) and in contrast, a high percentage (82%) who declared they call the doctor when their children are ill—could be explained by the social desirability effect as public health campaigns have lately been run in Romania to discourage self-medication.

The symptoms we found for which parents medicated their children were similar to those reported in other studies, i.e., fever, coughing, abdominal pain, and diarrhea [4,15,20].

The data of our research also showed that parents who administrate medicine without medical advice are mostly those who think medical services are difficult to access and whose children commonly have more than two health issues during the previous half-year. This result could be related to Romanian health care particularities, where primary care is under-used [21].

Our results are consistent with those of other authors showing that the most frequently parentally administered medicines are analgesics, antitussives, and antidiarrheals [4,15,22]. Recent studies conducted in adults show that medications for the musculoskeletal and nervous systems rank at the top (NSAIDs and other analgesics despite the risk of gastrointestinal complications), followed by medications for digestive, respiratory, and cardiovascular symptoms [17,23,24,25,26]. Parental reports of antibiotic self-administration to their children were not common in our study, despite the fact that the consumption of antibiotics in Romania is very high in the community; among the most used antibiotics are penicillins, cephalosporins, and quinolones [27]. We considered two possible explanations for the low rate of reported antibiotic self-medication to children. First, our parent group was highly educated, and they may recognize the risks of antibiotic overuse. Second, recognizing the societal harm from overuse of antibiotics, they may not have been fully honest in their responses to this question.

The high access to online information, as well as online healthcare communication sources, has not diminished parents’ focus on primary information sources, i.e., doctors and pharmacists, as highlighted by most studies [4,8,15]. The World Health Organization has acknowledged the importance and role of pharmacists in patients’ self-medication and self-care. The pharmacists’ role has become essential for responsible self-medication [28].

Accessing online sources to obtain medical information is becoming common practice among parents. Studies show that parents seeking information on their children’s symptoms and treatment via Google tend to be distrustful of medical assessment, requesting a second opinion, which often results in delayed diagnosis. Considering this aspect, they can also request a second opinion, but most importantly, they should discuss with the doctor the information they obtained from the internet. On the other hand, pediatricians should encourage parents to share their concerns in order to obtain answers about alternate diagnoses. Doctors’ knowledge of this approach, and its use thereof, during discussions with parents boosts the latter’s confidence level and subsequently the quality of the medical care [29].

This study provided insight into the pediatric self-medication patterns and beliefs among urban Romanian parents, an area for which little information was previously available in the published literature. This study reports on parental self-medication practices in a region of Romania that was not included in previous studies. Nevertheless, the present study has a number of limitations. The respondents are homogenous in respect to socio-demographic characteristics (age, education, residence, employment, number of children). Furthermore, other variables should be taken into account in order to achieve an integrated explanatory model on pediatric self-medication in a larger sample and assess the reliability of the questionnaire used in this study.

## 5. Conclusions

In Romania, we are still faced with a high incidence of self-medication, even within the pediatric population. The study highlighted the perceived risks of self-medication, as well as the beliefs and knowledge of this topic, and identified some determinants of parental predisposition to self-medication of their children. Implementing educational measures focused on parent communities is essential in our country.

## Figures and Tables

**Table 1:** Socio-demographic characteristics of the sample (*N* = 241).

		*N*	%
**Age**	18–25 years	7	3
	26–33 years	112	46
	34–41 years	101	42
	41–48	19	8
	+49	2	1
	Total	241	100
**Gender**	Male	26	11
	Female	215	89
	Total	241	100
**Marital Status**	Married	235	97
	Unmarried	4	2
	Divorced	2	1
	Total	241	100
**Number of Children Per Family**	1 child	144	59
	2 children	90	37
	3 children or more	7	3
	Total	241	100
**Education**	Baccalaureate	29	12
	University degree	162	67
	PhD or postgraduate studies	50	21
	Total	241	100

**Table 2:** Self-medication distribution across the frequency of children’s health issues.

Number of Children Health Issues In the Last 6 Months	How Many Times Did You Give Drugs to Your Children without Medical Advice?			Total	*p* * Value
	Often	Sometimes	Never		
less than 2	14 (7%)	126 (61%)	67 (32%)	207 (100%)	0.004
more than 2	8 (25%)	19 (59%)	5 (16%)	32 (100%)	
Total	22 (9%)	145 (61%)	72 (30%)	239 (100%)	

* Fisher’s Exact Test.

**Table 3:** Self-medication likelihood among parents who believe self-medication is allowed in case of minor health issues.

Self-Medication Is Allowed in Case of Children’s Minor Health Issues					
		Agree	Disagree	Total	*p* * Value
Parental medication of children without medical advice	yes	146 (92%)	12 (8%)	158 (100%)	
	no	49 (70%)	21 (30%)	70 (100%)	0.000
Total		195 (85%)	33 (15%)	228 (100%)	

* Fisher’s Exact Test.

**Table 4:** Self-medication likelihood among parents who believe self-medication is allowed in case of minor health issues.

Self-Medication Is a Solution When Health Services Are Difficult to Access						
		Agree	Disagree	Total	*p* * Value	
Parental medication of children without medical advice	yes	63 (44%)	79 (66%)	142 (100%)		
	no	13 (19)	54 (81%)	67 (100%)	0.000	
Total		76 (36%)	133 (64%)	209 (100%)		

* Fisher’s Exact Test.

**Table 5:** Self-medication likelihood among parents who believe self-medication is a solution when you do not have enough time.

Self-Medication Is a Solution When You Do Not Have Enough Time					
		Agree	Disagree	Total	*p* * Value
Parental medication of children without medical advice	yes	55 (37%)	93 (63%)	148 (100%)	
	no	15 (21%)	56 (79%)	71 (100%)	0.02
Total		70 (32%)	149 (68%)	219 (100%)	

* Fisher’s Exact Test.

**Table 6:** Source of the drugs administered to the children among parents who believe that self-medication is allowed in the case of minor health issues.

When Choosing to Handle Things on Your Own, How Do You Procure the Medicine?	Self-Medication Is Allowed in the Case of Minor Health Issues				Total
	System Missing	Agree	Disagree	Don’t Know/Don’t Answer	
directly from the pharmacy	1 (0.4%)	189 (81%)	32 (14%)	11 (5%)	22 (100%)
from acquaintances/relatives	1 (33%)	2 (67%)	0 (0%)	0 (0%)	146 (100%)
from previous prescriptions	0 (0%)	4 (80%)	1 (20%)	0 (0%)	73 (100%)
Total	2 (0.8%)	195 (81%)	33 (14%)	11 (5%)	241 (100%)

## Supplementary Materials

The following are available online at https://www.mdpi.com/1010-660X/56/6/312/s1, Text S1: Questionare for self-medication among the pediatric population.
