# Supplementary material for: Patterns and Factors Associated with Self-Medication among the Pediatric Population in Romania
Source: Medicina (Kaunas). 2020 Jun 25;56(6):312. doi: 10.3390/medicina56060312 (PMC7353868; doi:10.3390/medicina56060312)
Supplement: Supplementary file 1 [file medicina-56-00312-s001.pdf]

Dear Madam/Sir
We invite you to take part in a survey regarding the attitudes and behaviours of parents toward the administration of medical treatments.
The data collected in this study will be used for publications in scientific journals, in research reports and in a doctoral thesis. We mention that we do NOT request personal data (name, address, pone number) and our study has complied with the Romanian legislation (Law 190/2018) and GDPR - The General Data Protection Regulation 679/2016.

Your answers are anonymous and confidential. We ensure the confidentiality of the answers.

Completing this online study is voluntary and it takes approximately 15-20 minutes.

If there are any reasons you are unable to complete the questionnaire, you may withdraw.

By clicking on the button below, you confirm your participation in this study, but also the fact that you are at least 18 years old and that you have voluntarily chosen to complete the questionnaire.

*I agree to answer to the questionnaire.*

*I do not agree; I do not want to participate in this research.*

1. Your age:
------

2. Sex *
Male
Female

3. Marital status: *
Married
Single
Divorced
Widow

4. Studies *
Without school
General school
High school - Bachelor's degree
Post-secondary studies
Higher education
Doctoral / postgraduate studies
Vocational school

5. Do you currently have a job? *
Yes
No

6. Your working schedule is: *
Full time (8h)
Half-time (part time-4h)
Special program (work from home)

7. How many children do you have? *
1 child

2 children
3 or more than 3 children

8. How often have you been treating your children without your doctor's advice?
Often
Sometimes
Never

9. In the last 6 months, how many times your child has had a health problem:
Never
Once
2-3 times
4-5 times
More than 5 times

10. To what extent do you resort to the following situations when the child is ill:

| | To a large extent | To a small extent | Never |
|---|---|---|---|
| Go to the hospital | ☐ | ☐ | ☐ |
| Call the known paediatric telephone lines | ☐ | ☐ | ☐ |
| Get in touch with a trusted acquaintance | ☐ | ☐ | ☐ |
| Call your child's paediatrician / family doctor | ☐ | ☐ | ☐ |
| Give medicine to your child | ☐ | ☐ | ☐ |
| Give phytoterapies | ☐ | ☐ | ☐ |
| Give homeopathic remedies | ☐ | ☐ | ☐ |
| Search on Google | ☐ | ☐ | ☐ |
| Search only on trusted medical online sources | ☐ | ☐ | ☐ |
| Resume the previous treatments/ schemes / prescriptions from your doctor | ☐ | ☐ | ☐ |
| Ask about treatment on online parenting groups | ☐ | ☐ | ☐ |

11. What are the situations in which you deal with your child's problem alone?
Fever
Cough
Vomiting
Abdominal pain
Diarrhoea
Minor trauma

Other. Which?....................

12. What medicines do you give to your child when dealing with his or her health problem?
Analgesics
Antipyretics (paracetamol, ibuprofen etc.)
Antibiotics
Antitussive
Antidiarrheal
Other. Which?................

13. From what age of your child have you managed alone certain health problems?
Less than 6 months
6-12 months
13-24 months
After 24 months

14. How do you get the drugs used in your child's health problems?
Immediately from the pharmacy
You call on an acquaintance / relative / friend
You use the previous prescriptions

15. When and to what extent do you decide to treat alone your child in the following situations?

| | To a large extent | To a small extent | Never |
|---|---|---|---|
| At the end of the week | ☐ | ☐ | ☐ |
| When you are out of town | ☐ | ☐ | ☐ |
| During the night | ☐ | ☐ | ☐ |
| When you cannot access medical services | ☐ | ☐ | ☐ |

16. When do you decide to self-administer medications to your child?
When the first symptoms appear
When you recognize the symptoms of a previous illness
When the child's condition worsens

17. The medication information you obtain from:
Mass media (TV, newspapers, magazines, etc.)
Friends
Pharmacist
Doctor
Google / Internet search
Online groups of parents from forums and social media

Specialized medical sites
Other

18. Do you think you are exposing your child to a risk by administering medicines without medical advice?
Yes
No

19. If yes, which of the following situations represent possible sources of risk:

Inappropriate medication
Inappropriate dose
Side effects of medicines
Misdiagnosis of severe illnesses
Delayed presentation to the doctor
Drug interactions

20. To what extent do you agree with the following statements:

| | Agree | Disagree | Don't know/I don't answer |
|---|---|---|---|
| Self-medication is a solution when you don't have time | ☐ | ☐ | ☐ |
| Self-medication is more effective when the medical advice / consultation is difficult to access | ☐ | ☐ | ☐ |
| Self-medication is cheaper than medical consultation | ☐ | ☐ | ☐ |
| Self-medication is allowed in case of minor health issues | ☐ | ☐ | ☐ |

21. How often have you searched for health information on the Internet?
Always
Often
Rare
Never

22. How often have you read the following on the Internet?

| | Always | Often | Rarely | Never |
|---|---|---|---|---|
| Treatment of certain paediatric diseases | □ | □ | □ | □ |
| Symptoms related to specific diseases | □ | □ | □ | □ |
| The stories of parents who have encountered children's health problems | □ | □ | □ | □ |
| Natural methods of treating children's diseases - phytotherapy | □ | □ | □ | □ |
| Homeopathic remedies for treating children's diseases | □ | □ | □ | □ |

23. In your opinion, to what extent did the information you read on the Internet help you in your child's health problem?
To a large extent
To a small extent
I don't know / I'll not answer

24. Do you have a Facebook, Instagram, Twitter account?
Yes
Not

25. Are you a member of an online medical discussion group?
Yes
Not
